# Efficacy and safety of concurrent chemoradiotherapy in ECOG 2 patients with locally advanced non-small-cell lung cancer: a subgroup analysis of a randomized phase III trial

**DOI:** 10.1186/s12885-020-06780-x

**Published:** 2020-04-06

**Authors:** Nan Bi, Lipin Liu, Jun Liang, Shixiu Wu, Ming Chen, Changxing Lv, Lujun Zhao, Anhui Shi, Wei Jiang, Yaping Xu, Zongmei Zhou, Jingbo Wang, Wenqing Wang, Dongfu Chen, Zhouguang Hui, Jima Lv, Hongxing Zhang, Qinfu Feng, Zefen Xiao, Xin Wang, Tao Zhang, Weibo Yin, Junling Li, Jie He, Luhua Wang

**Affiliations:** 1 Department of Radiation Oncology, National Cancer Center/National Clinical Research Center for Cancer/Cancer Hospital, Chinese Academy of Medical Sciences and Peking Union Medical College, No. 17 Panjiayuannanli, Chaoyang District, Beijing, 100021 China; 2 Department of Radiation Oncology, The First Affiliated Hospital of Wenzhou Medical University, Wenzhou, China; 3 Department of Radiation Oncology, Sun Yat-sen University Cancer Center, Guangzhou, China; 4 Department of Radiation Oncology, Shanghai Chest Hospital, Shanghai, China; 5 Department of Radiation Oncology, Tianjin Cancer Hospital, Tianjin, China; 6 Department of Radiation Oncology, Beijing Cancer Hospital, Beijing, China; 7 Department of Radiation Oncology, Zhongshan Hospital Fudan University, Shanghai, China; 8 Department of Radiation Oncology, Zhejiang Cancer Hospital, Hangzhou, China; 9 Department of Medical Oncology, National Cancer Center/Cancer Hospital, Chinese Academy of Medical Sciences and Peking Union Medical College, Beijing, China; 10 Department of Thoracic Surgery, National Cancer Center/Cancer Hospital, Chinese Academy of Medical Sciences and Peking Union Medical College, Beijing, China; 11 Department of Radiation Oncology, National Cancer Center/ Cancer Hospital & Shenzhen Hospital, Chinese Academy of Medical Sciences and Peking Union Medical College, No. 113 Baohedadao, Longgang District, Shenzhen, 518116 China

**Keywords:** Locally advanced, Non-small-cell lung cancer, ECOG 2, Chemoradiotherapy, Efficacy, Toxicity

## Abstract

**Background:**

There is no consensus on the therapeutic approach to ECOG 2 patients with locally advanced non-small-cell lung cancer (LA-NSCLC), despite the sizable percentage of these patients in clinical practice. This study focused on the efficacy, toxicity and the optimal chemotherapy regimen of CCRT in ECOG 2 patients in a phase III trial.

**Methods:**

Patients capable of all self-care with bed rest for less than 50% of daytime were classified as ECOG 2 subgroup. A subgroup analysis was performed for ECOG 2 patients recruited in the phase III trial receiving concurrent EP (etoposide + cisplatin)/PC (paclitaxel + carboplatin) chemotherapy with intensity-modulated radiation therapy (IMRT) or three-dimensional conformal external beam radiation therapy (3D-CRT).

**Results:**

A total of 71 ECOG 2 patients were enrolled into the study. Forty-six (64.8%) patients were treated with IMRT technique. The median overall survival (OS) and progression free survival (PFS) for ECOG 2 patients were 16.4 months and 9 months, respectively. No difference was observed in treatment compliance and toxicities between ECOG 2 patients and ECOG 0–1 patients. Within the ECOG 2 group (31 in the EP arm and 40 in the PC arm), median OS and 3-year OS were 15.7 months and 37.5% for the EP arm, and 16.8 months and 7.5% for the PC arm, respectively (*p* = 0.243). The incidence of grade ≥ 3 radiation pneumonitis was higher in the PC arm (17.5% vs. 0.0%, *p* = 0.014) with 5 radiation pneumonitis related deaths, while the incidence of grade 3 esophagitis was numerically higher in the EP arm (25.8% vs. 10.0%, *p* = 0.078).

**Conclusions:**

CCRT provided ECOG 2 patients promising outcome with acceptable toxicities. EP might be superior to PC in terms of safety profile in the setting of CCRT for ECOG 2 patients. Prospective randomized studies based on IMRT technique are warranted to validate our findings.

**Trial registration:**

ClinicalTrials.gov registration number: NCT01494558. (Registered 19 December 2011).

## Background

Non-small-cell lung cancer (NSCLC) accounts for 85% of all lung cancers [1], and approximately 30% of NSCLC present with locally advanced disease (LA-NSCLC) [2]. Performance status (PS) is a recognized prognostic factor for lung cancer which is often taken into account while choosing therapeutic strategy [3]. The Eastern Cooperative Oncology Group (ECOG) scale is the most commonly used tool to assess PS, with scores ranging from 0 (normal functional status) to 5 (death) [4]. Typically, patients with an ECOG score of 0–1 are labeled as “good PS”. For LA-NSCLC patients with good PS, concurrent chemoradiotherapy (CCRT) is the standard-of-care [5].

A pooled analysis demonstrated that approximately 30% of lung cancer patients had an ECOG score of 2 [6]. Despite a sizable percentage of ECOG 2 patients, no specific treatment guidelines exist for this subgroup and management options in clinical practice range from radiotherapy/chemotherapy alone to combined modality of radiotherapy and chemotherapy. In the clinical trials evaluating CCRT, patients with ECOG score of 2 suggesting slightly poorer treatment tolerance and prognosis have been excluded or underrepresented [7–9]. As a result, the efficacy and safety of CCRT for ECOG 2 patients with LA-NSCLC remains to be defined.

In the modern era, three-dimensional conformal radiation therapy (3D-CRT) and subsequently to intensity-modulated radiation therapy (IMRT) offer further improvements in conformality. Recently, IMRT has been demonstrated to improve dosimetry, reduce the risk of radiation induced toxicities, and at least provide equivalent disease related outcome compared to three-dimensional conformal external beam radiotherapy (3D-CRT) [10]. The clinical benefit brought by utilization of IMRT may bring opportunities of definitive treatment for ECOG 2 patients.

The phase III trial [11] which compared efficacy of concurrent thoracic radiotherapy with either etoposide/cisplatin (EP) or carboplatin/paclitaxel (PC) in LA-NSCLC revealed that EP might be superior to weekly PC in terms of overall survival (OS). In contrast to other phase III trials, this trial enrolled ECOG 2 patients with a higher proportion at approximately 40%. Since limited treatment outcome data of CCRT have been available for ECOG 2 patients with LA-NSCLC, we present the data from a subgroup analysis of the phase III trial above that focused on the efficacy, toxicity and the optimal chemotherapy regimen of CCRT in ECOG 2 patients with LA-NSCLC.

## Methods

The trial was a prospective, randomized, open, multicenter phase III study comparing the efficacy and safety of concurrent EP versus PC chemotherapy with radiotherapy for LA-NSCLC. Patients were stratified by institution and stage before randomization. The Ethics Committee of the participating institutions approved the study protocol, and all patients provided signed informed consent before enrollment.

### Patient eligibility

Patients eligible for the phase III trial had histologically/cytologically confirmed inoperable AJCC stage III NSCLC. Eligibility criteria included ECOG≤2; unintended weight loss≤10%; forced expiratory volume in 1 s (FEV1) ≥40% of normal; adequate bone marrow, renal, and hepatic function; and absence of malignant pleural effusion, active uncontrolled infection, significant cardiovascular disease, history of other malignancies and previous treatment with radiotherapy or chemotherapy.

### Treatment

The chemotherapy regimen for the EP arm consisted of etoposide 50 mg/m^2^ on days 1–5 and cisplatin 50 mg/m^2^ on days 1, 8, every 4 weeks for two cycles; and chemotherapy regimen for the PC arm consisted of 45 mg/m^2^ paclitaxel and carboplatin (AUC 2) on day 1 once a week. Radiation regimen was 2 Gy per fraction to a target dose of 60 to 66 Gy using 3D-CRT or simplified IMRT.

### Evaluation and follow-up

Pre-treatment assessment included chest and abdominal CTs, brain MRI/CTs, bronchoscopies, and radionuclide bone scans. The follow-up evaluations consisted of patient history, a physical examination, and chest CT at intervals of 3 months for 2 years and then 6 to 12 months for 3 years, then annually. Other imaging examinations were obtained as clinically indicated.

The treatment response was evaluated using the Response Evaluation Criteria in Solid Tumors (RECIST) version 1.0. Toxicities were graded according to the Common Toxicity Criteria for Adverse Events (CTCAE) version 3.0.

### Definition of ECOG 2 subgroup and study aims

The ECOG PS scale is a 6-point numerical scale, with scores ranging from 0 (normal functional status) to 5 (death), in incremental steps of 1. In accordance with the ECOG scale [4], we classified patients capable of all self-care with bed rest for less than 50% of daytime as ECOG 2 subgroup.

The aims of the present subgroup analyses were (1) explore the efficacy and safety of concurrent chemoradiotherapy for ECOG 2 patients with LA-NSCLC and (2) identify the optimal chemotherapy regimen concurrent with radiation for the ECOG 2 subgroup.

### Statistical analysis

OS, progression free survival (PFS) and cancer specific survival (CSS) were defined from the date of randomization to the time of specific event: any cause of death, progression, or cancer specific death. The date of death was chosen as the date of progression if no other information on progression was documented. OS and PFS analyses were performed using the Kaplan-Meier method and the log-rank test. Cox proportional hazards models, stratified by age, sex, pathology, weight loss, stage and smoking history were used to estimate hazard ratios (HRs) and 95% confidence intervals (CIs). A competing risk survival analysis was conducted for CSS using Fine and Gray’s method [12]. Dichotomous data were compared by chi-square test and continuous variables were compared using Mann-Whitney U test. A two-sided *p* < 0.05 was considered as statistically significant. All data were processed by SPSS software version 19.0 or R version 3.5.1 (http://www.R-project.org/).

## Results

### Patient characteristics

Two hundred patients were enrolled from nine institutions in China from August 2007 to August 2011. Of the 200 patients, nine patients were excluded and three had stage IV disease. Two patients had small cell lung cancer and 4 refused to be randomized. 191 participants (95 in EP arm and 96 in PC arm) were treated according to protocol and eligible for analysis. The characteristics of the 191 patients are presented in Table 1.

**Table 1:** Demographic and baseline clinical characteristics of patients

Patient characteristic	ECOG 2 group			ECOG 0–1 group			***P***^**b**^
	EP arm (***n*** = 31)	PC arm (***n*** = 40)	***p***^**a**^	EP arm (***n*** = 64)	PC arm (***n*** = 56)	***p***^**a**^	
Age			0.746			0.403	0.923
< 65, y	23 (74.2%)	31 (77.5%)		51 (79.7%)	41 (73.2%)		
≥ 65, y	8 (25.8%)	9 (22.5%)		13 (20.3%)	15 (26.8%)		
Median	56	58.5		59	56		
Range	32–70	42–70		33–70	39–70		
Gender			0.627			0.382	0.308
Male	25 (80.6%)	34 (85.0%)		55 (85.9%)	51 (91.1%)		
Female	6 (19.4%)	6 (15.0%)		9 (14.1%)	5 (8.9%)		
Weight loss			0.276			0.878	0.674
< 5%	17 (54.8%)	27 (67.5%)		42 (65.6%)	36 (64.3%)		
≥ 5%	14 (45.2%)	13 (32.5%)		22 (34.4%)	20 (35.7%)		
Smoking history			0.887			0.644	0.457
Yes	22 (71.0%)	29 (72.5%)		48 (75.0%)	44 (78.6%)		
No	9 (29.0%)	11 (27.5%)		16 (25.0%)	12 (21.4%)		
Pathology			0.912			0.500	0.082
Squamous	23 (74.2%)	31 (77.5%)		42 (65.6%)	31 (55.4%)		
Adenocarcinoma	6 (19.4%)	6 (15.0%)		14 (21.9%)	15 (26.8%)		
Other	2 (6.5%)	3 (7.5%)		8 (12.5%)	10 (17.9%)		
AJCC stage			0.747			0.566	0.326
IIIA	6 (19.4%)	9 (22.5%)		19 (29.7%)	14 (25.0%)		
IIIB	25 (80.6%)	31 (77.5%)		45 (70.3%)	42 (75.0%)		
Tumor stage			0.612			0.392	0.662
T1	1 (3.2%)	0 (0.0%)		0 (0.0%)	0 (0.0%)		
T2	19 (61.3%)	22 (55.0%)		41 (64.1%)	33 (58.9%)		
T3	7 (22.6%)	13 (32.5%)		17 (26.6%)	13 (23.2%)		
T4	4 (12.9%)	5 (12.5%)		6 (9.4%)	10 (17.9%)		
Nodal stage			0.594			0.564	0.409
N2	9 (29.0%)	14 (35.0%)		23 (35.9%)	23 (41.1%)		
N3	22 (71.0%)	26 (65.0%)		41 (64.1%)	33 (58.9%)		
Pre-RT pulmonary function							
FEV_1_ (L)^c^	2.09 (1.08–4.38)	1.99 (1.17–2.98)	0.638	2.23 (1.15–4.05)	2.07 (0.84–3.08)	0.266	0.277
FEV_1_ (% predicted) ^c^	65.1% (35.6–117.1%)	65.5% (42.4–103.6%)	0.656	70.3% (39.3–110.6%)	63.1% (22.3–96.7%)	0.133	0.607

*Abbreviations*: *EP* etoposide/cisplatin, *PC* paclitaxel/carboplatin, *ECOG* Eastern Cooperative Oncology Group, *AJCC* American Joint Committee on Cancer, *FEV*_*1*_ forced expiratory volume in 1 s

^a^*p* value for testing the null hypothesis of no difference between patients receiving EP and PC chemotherapy

^b^*p* value for testing the null hypothesis of no difference between ECOG 2 group and ECOG 0–1 group

^c^ Median (range)

A total of 71 ECOG 2 patients were enrolled into the study, accounting for almost 40% of all patients. The median age of the ECOG2 patients was 58 years (range, 32–70 years). The majority of patients were younger than 65 years old (76.1%) and male (83.1%) with no significant (< 5%) weight loss (62.0%) and a smoking history (71.8%). The most common pathology subtype was squamous cell carcinoma (SCC) (76.1%). And 78.9% of patients presented with stage IIIB disease. As shown in Table 1, no statistically significant differences were found in the clinical characteristics between the ECOG 2 and the ECOG 0–1 subgroups. Among ECOG 2 patients, 31 patients were assigned to the EP arm and 40 to the PC arm. Clinical characteristics were generally well balanced between the two treatment arms within the ECOG 2 group.

### Treatment delivery

As shown in Table 2, radiotherapy was administered according to protocol in 97.2% ECOG 2 patients, with 1 patient in the EP arm refused to complete full-dose radiotherapy and 1 patient in the PC arm didn’t finish radiotherapy due to toxicity. A total of 46 (64.8%) ECOG 2 patients were treated with IMRT technique. 78.9% of ECOG 2 patients received a radiotherapy dose of ≥60 Gy. Regarding chemotherapy compliance for ECOG 2 patients, more patients in the EP arm (90.3%) completed concurrent treatment as planned than those in the PC arm (60.0%) (*p* = 0.004). The main reason for not completing chemotherapy was unacceptable toxicity, which was seen in 2 patients and 13 patients in the EP and PC arms, respectively.

**Table 2:** Treatment delivery and reasons for treatment discontinuation

Variable	ECOG 2 group			ECOG 0–1 group			***p***^**b**^
	EP arm (***n*** = 31)	PC arm (***n*** = 40)	***p***^**a**^	EP arm (***n*** = 64)	PC arm (***n*** = 56)	***p***^**a**^	
Radiotherapy							
Radiotherapy			0.747			0.268	0.113
≥ 60 Gy	25 (80.6%)	31 (77.5%)		54 (84.4%)	51 (91.1%)		
< 60 Gy	6 (19.4%)	9 (22.5%)		10 (15.6%)	5 (8.9%)		
GTV (cm^3^) ^c^	98.0 (27.3–383.3)	123.1 (56.4–298.6)	0.859	123.9 (20.6—307.7)	111.1 (8.6–485.4)	0.809	0.797
Mean lung dose (cGy) ^c^	1591 (900–1891)	1550 (970–2004)	0.657	1576 (957–2100)	1588 (969–1895)	0.531	0.908
V20 of the both lungs (%) ^c^	26 (20–32)	27 (13–35)	0.292	27 (14–35)	25.5 (14–31)	0.127	0.176
Reason for radiotherapy discontinuation							
Unacceptable toxicity	0	1		1	0		
Comorbidity	0	0		0	0		
Patients request	1	0		0	1		
Chemotherapy							
Concurrent chemotherapy			0.004			0.011	0.788
EP = 2 cycles or PC ≥ 5 weeks	28 (90.3%)	24 (60.0%)		54 (84.4%)	36 (64.3%)		
EP < 2 cycles or PC < 5 weeks	3 (9.7%)	16 (40.0%)		10 (15.6%)	20 (35.7%)		
Reason for concurrent chemotherapy discontinuation							
Unacceptable toxicity	2	13		10	19		
Comorbidity	0	1		0	0		
Patients request	1	2		0	1		
Consolidation Chemotherapy			0.594			0.080	< 0.001
Yes	7 (22.6%)	7 (17.5%)		41 (64.1%)	27 (48.2%)		
No	24 (77.4%)	33 (82.5%)		23 (35.9%)	29 (51.8%)		

*Abbreviations*: *EP* etoposide/cisplatin, *PC* paclitaxel/carboplatin, *ECOG* Eastern Cooperative Oncology Group, *GTV* gross tumor volume

^a^*p* value for testing the null hypothesis of no difference between patients receiving EP and PC chemotherapy

^b^*p* value for testing the null hypothesis of no difference between ECOG 2 group and ECOG 0–1 group

^c^ Median (range)

In terms of radiotherapy technique, more ECOG 2 patients were treated with IMRT than ECOG 0–1 patients (64.8% vs. 34.2%, *p* < 0.001). After CCRT, a significantly smaller percentage of ECOG 2 patients (19.7%) received consolidation chemotherapy than that in ECOG 0–1 patients (56.7%) (*p* < 0.001). No significant difference was observed in terms of radiotherapy discontinuation、radiation dose、gross tumor volume (GTV) and dosimetric parameters (mean lung dose and V20) between the ECOG 0–1 and ECOG 2 groups, or EP and PC arms within the ECOG 2 group (Table 2).

### Efficacy

**Fig. 1:**
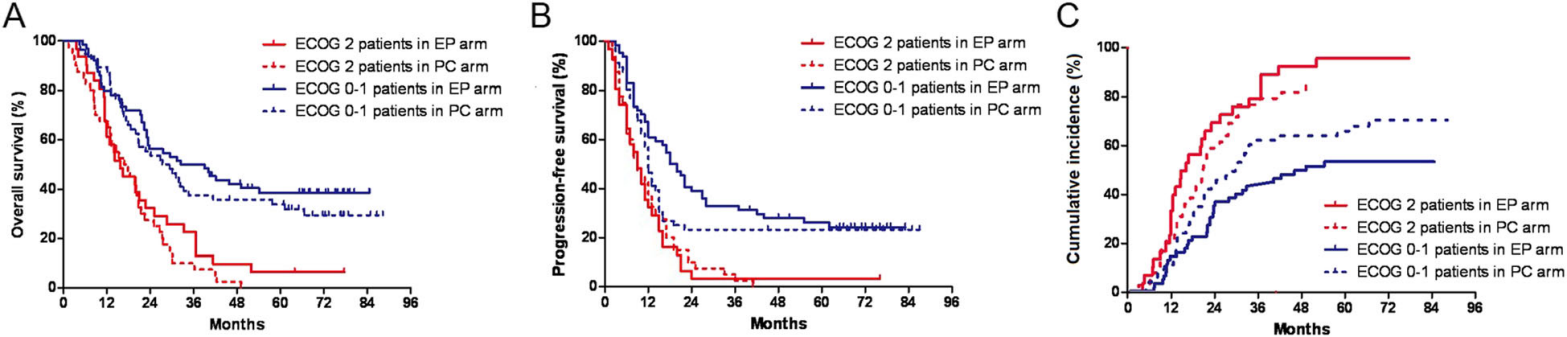
**a**-**b**, Kaplan-Meier curves by arm and ECOG status for overall survival (**a**) and progression-free survival (**b**). **c**, Cumulative incidence function of cancer death from competing risk survival analysis by arm and ECOG status. *P* values were from log-rank tests for **a** and **b**, and from Fine and Gray’s method for **c**. PC = paclitaxel/carboplatin; EP = etoposide/cisplatin; ECOG = Eastern Cooperative Oncology Group performance score

As shown in Fig. 1, ECOG 0–1 patients achieved significantly better OS compared with ECOG 2 patients (median OS, 30.1 months vs. 16.4 months; 3-year OS, 44.2% vs. 15.5%; *p* < 0.001). Consistent with the OS results, the median PFS and 3-year PFS for ECOG 0–1 patients (14 months and 28.3%) were also superior to those for the ECOG 2 patients (9 months and 2.8%) (*p* < 0.001). Considering the non-cancer related death as a competing risk, competing risk survival for the CSS was performed. The 3-year cumulative incidence of cancer death for the ECOG 0–1 patients (50.8%) was significantly lower than that for the ECOG 2 patients (76.1%) (*p* < 0.001). For ECOG 2 patients, median OS and 3-year OS were 15.7 months and 37.5% for the EP arm and 16.8 months and 7.5% for the PC arm (*p* = 0.243). Median PFS and 3-year PFS were 9.0 months and 3.2% for the EP arm and 9.0 months and 2.5% for the PC arm (*p* = 0.709). There was no difference in 3-year cumulative incidence of cancer death between EP and PC arm (77.4% vs. 75.0; *p* = 0.276).

Objective response rate (ORR) did not differ between the ECOG 0–1 and ECOG 2 patients (71.7% vs. 64.8%, *p* = 0.320). Of the 71 ECOG 2 patients, the responses of complete response (CR)、partial response (PR) and stable disease (SD) were observed in 1 (1.4%) patients, 45 (63.4%) patients and 25 (35.2%) patients, respectively. The ORR was 67.7% (with 0% CR) in the EP arm versus 62.5% (with 2.5% CR) in the PC arm without a significant difference (*p* = 0.646).

A total of 184 patients (64 with ECOG 2 and 120 with ECOG 0–1) were available for patterns of first failure analysis. A significant difference in treatment failure pattern was seen between the ECOG 0–1 and ECOG 2 patients (*p* < 0.001). The incidence of locoregional failure for ECOG 2 patients was much higher than that for ECOG 0–1 patients (48.3% vs. 15.8%). A smaller percentage of ECOG 2 patients had brain metastasis as first relapse (2.8% vs. 14.2%). Within the ECOG 2 patients, the EP arm and the PC arm showed similar patterns of first failure with no significant differences.

**Fig. 2:**
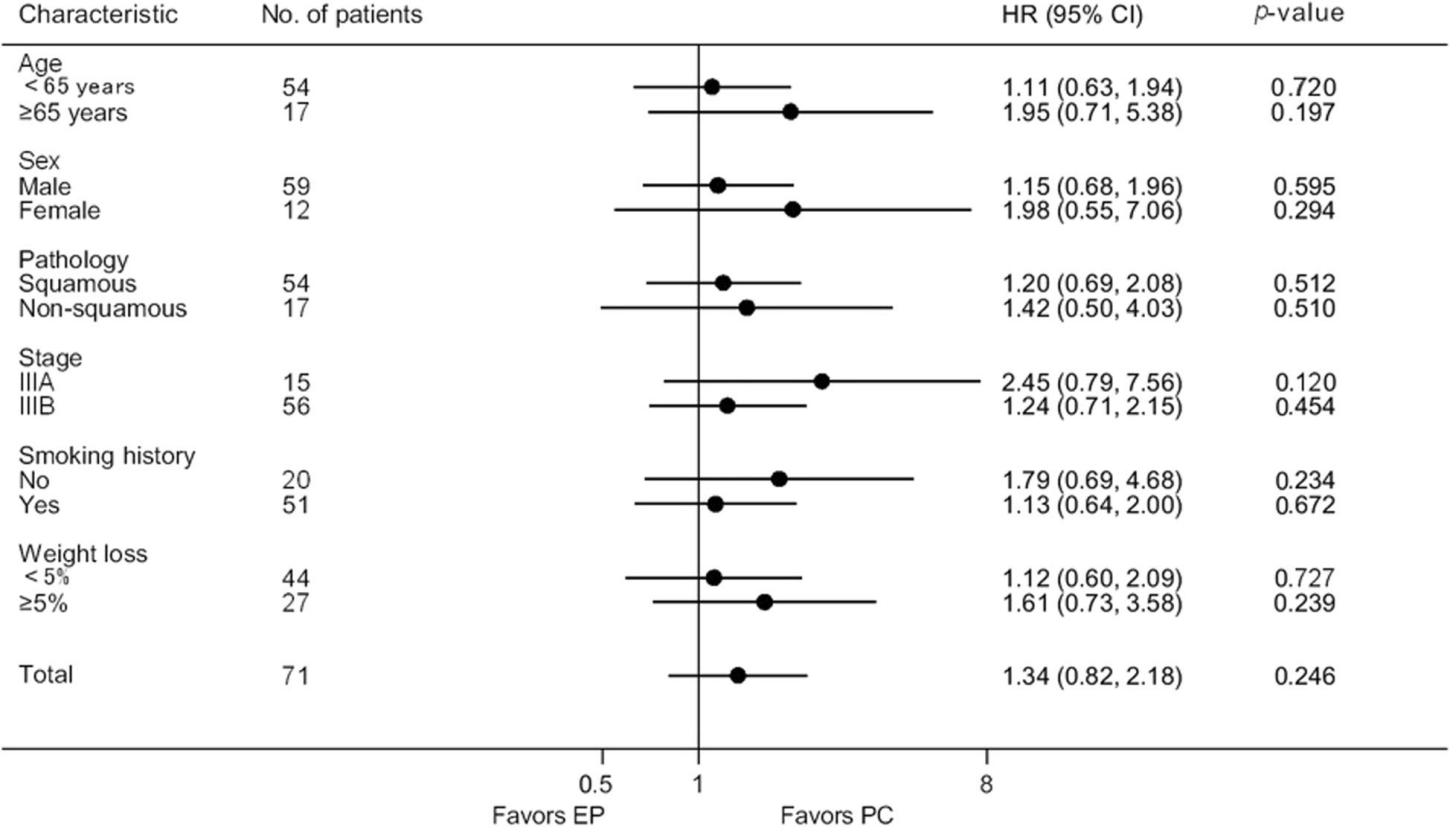
Forest plot of HRs for overall survival by prognostic factors. PC = paclitaxel/carboplatin; EP = etoposide/cisplatin; HR = hazard ratio; CI = confidence interval

A subgroup analysis was performed to evaluate whether there was a differential effect of different chemotherapy regimen in predefined subgroups of ECOG 2 patients. As shown in Fig. 2, there was no difference in OS between the EP arm and the PC arm in any subgroups analyzed.

### Toxicity

As shown in Table 3, there was no significant difference regarding hematologic toxicities、esophagitis、radiation pneumonitis、gastrointestinal or dermatological toxicities between ECOG 0–1 and ECOG 2 patients. For ECOG 2 patients, a significantly higher portion of patients developed grade ≥ 3 radiation pneumonitis in the PC arm (17.5%) than those in EP arm (0.0%) (*p* = 0.014). 5 (7%) ECOG 2 patients in the PC arm died from grade 5 radiation pneumonitis. The incidence of grade 3 esophagitis was numerically higher in the EP arm (25.8%) than that in the PC arm (10.0%), though not reaching statistical significance (*p* = 0.078). No significant difference in hematologic toxicities, gastrointestinal toxicities or dermatological toxicities between the two treatment arms was observed.

**Table 3:** Toxicity according to performance status and treatment

Toxicity	ECOG 2 group			ECOG 0–1 group			***p***^**b**^
	EP arm (***n*** = 31)	PC arm (***n*** = 40)	***p***^**a**^	EP arm (***n*** = 64)	PC arm (***n*** = 56)	***p***^**a**^	
Hematological			0.663			0.725	0.854
Grade 3/4	10 (32.3%)	11 (27.5%)		19 (29.7%)	15 (26.8%)		
Grade 1/2	21 (67.7%)	29 (72.5%)		45 (70.3%)	41 (73.2%)		
Esophagitis			0.078			0.017	0.230
Grade 3	8 (25.8%)	4 (10.0%)		11 (17.2%)	2 (3.6%)		
< Grade 3	23 (74.2%)	36 (90.0%)		53 (82.8%)	54 (96.4%)		
Radiation pneumonitis			0.014			0.066	0.428
≥ Grade 3	0 (0.0%)	7 (17.5%)		7 (10.9%)	1 (1.8%)		
< Grade 3	31 (100.0%)	33 (82.5%)		57 (89.1%)	55 (98.2%)		
Gastrointestinal toxicity			0.327			0.285	0.950
≥ Grade 3	3 (9.7%)	8 (20.0%)		8 (12.5%)	11 (19.6%)		
< Grade 3	28 (90.3%)	32 (80.0%)		56 (87.5%)	45 (80.4%)		
Dermatological toxicity						0.598	0.296
≥ Grade 3	0 (0.0%)	0 (0.0%)		1 (1.6%)	2 (3.6%)		
< Grade 3	31 (100.0%)	40 (100.0%)		63 (98.4%)	54 (96.4%)		

*Abbreviations*: *EP* etoposide/cisplatin, *PC* paclitaxel/carboplatin, *ECOG* Eastern Cooperative Oncology Group

^a^*p* value for testing the null hypothesis of no difference between patients receiving EP and PC chemotherapy

^b^*p* value for testing the null hypothesis of no difference between ECOG 2 group and ECOG 0–1 group

## Discussion

As a widely recognized prognostic factor for lung cancer, PS has a significant impact on treatment choice. While many phase III trials have established CCRT as a standard care for LA-NSCLC with good PS, the best treatment approach for ECOG 2 patients has yet to be determined. Patients with poor prognostic factors including age ≥ 70 years, ECOG≥2, weight loss > 5% or 10% or presence of major comorbidities were referred to in the literature as “poor risk”. A few prospective trials have investigated proper treatment modality for poor risk patients.

Two phase II studies [13, 14] conducted by Southwest Oncology Group (SWOG) evaluated CCRT approach for poor risk stage III NSCLC, in which the percentages of ECOG 2 patients were 18% (*n* = 11) and 43% (*n* = 37) respectively. Patients were treated with carboplatin/etoposide chemotherapy given concurrently with two-dimensional radiotherapy of curative dose (61 Gy). The results suggested that CCRT was well tolerated and yielded a promising survival (median OS, 13 months and 10.2 months) comparable to that of patients with better prognosis receiving sequential CRT reported in contemporary studies [15, 16]. Based on the encouraging outcome achieved in the above single arm phase II trials, many clinical trials have investigated whether CCRT is superior to radiotherapy alone or chemotherapy alone for poor risk stage III NSCLC. Nawrocki et al. [17] conducted a phase II study which randomly assigned poor-risk stage III NSCLC to either radiation alone of palliative dose (30Gy) or the same radiation dose delivered concurrently with the third of 3 cycles of cisplatin/vinorelbine. Three-dimensional conformal planning was used. This trial enrolled 12 (25%) ECOG 2 patients in the radiotherapy arm and 14 (27%) in the concurrent chemoradiation arm. The study demonstrated that concurrent chemotherapy significantly prolonged median OS (9 months vs. 12.9 months), 1-year OS (25% vs. 57%) and 2-year OS (6% vs. 24%) at the expense of worsened hematological toxicities. A Norwegian multicenter phase III trial [18] compared concurrent carboplatin/vinorelbine and palliative thoracic radiation (42 Gy/15 fractions) with chemotherapy alone for poor-risk stage III NSCLC. The study concluded that CCRT was superior to chemotherapy alone with respect to survival and quality of life. There were 20.2% (*n* = 19) ECOG 2 patients in the chemotherapy arm and 23.3% (*n* = 21) in the CCRT arm. Subgroup analysis of ECOG 2 patients revealed that median OS was similar in both treatment arms (7.8 months in the CCRT arm and 7.5 months in the chemotherapy arm), possibly because of the small sample size (*p* = 0.24), though 1-year survival rate was much higher numerically in the CCRT arm (28.6%) than in the chemotherapy arm (10.5%).

In our phase III trial, good PS was a favorable prognostic factor for survival. The median OS was 30.1 months versus 16.4 months for the ECOG 0–1 arm versus the ECOG 2 arm (*p* < 0.001). The encouraging median OS of 16.4 months for the ECOG 2 patients was better than the outcome data for either good PS patients receiving sequential CRT (median OS 11 months to 14.6 months), or poor risk patients receiving CCRT (median OS 10.2 months to 14 months) reported in randomized clinical trials [13, 19, 20]. The prolonged survival of ECOG 2 patients conferred by CCRT may be attributed to several reasons as follows. Firstly, CCRT is superior to sequential chemoradiotherapy theoretically given the spatial cooperation and radiosensitizing properties of concurrent chemotherapy [21]. Secondly, except for PS of ECOG 2 and weight loss ≥5% (*n* = 27), our enrolled patients had no other poor prognostic factors. As a result, the prognosis of ECOG 2 patients in our study was more favorable than that of the poor risk patients enrolled in other clinical trials [13, 14, 17, 18, 20]. Thirdly, our CCRT intensity including RT dose and chemotherapy regimen was more aggressive than that administered for poor risk patients with palliative intent [17, 18]. In our study, CCRT was tolerated well in ECOG 2 patients with no significant increase in toxicities compared with good PS patients. The increased therapeutic intensity may result in the prolonged survival in our study than that achieved in palliative setting. Lastly, unlike historical studies using two-dimensional RT or 3D-CRT to treat poor risk patients, our study implemented IMRT for 64.8% ECOG 2 patients which may contribute to improved survival compared to historical results. The survival benefit conferred by IMRT planning has been reported in the population-based results from SEER and National Cancer Database [11, 22] comparing IMRT versus 3D-CRT.

In routine oncologic practice, LA-NSCLC patients with poor PS are often not candidates for standard CCRT due to poor tolerance and increased toxicities. However, our study suggested that treatment compliance and toxicities were similar between the ECOG 0–1 patients and the ECOG 2 patients. Radiation technique development and better supportive care have brought opportunities of definitive treatment for selective patients with poor performance status. Compared with 3D-CRT, IMRT has been reported to reduce treatment-related toxicities including esophageal and pulmonary toxicity [23, 24]. In addition, employing timely supportive care made acute toxicities manageable in order to avoid treatment interruptions and discontinuations. In our study, ECOG 2 patients were less likely to receive consolidation chemotherapy than ECOG 0–1 patients. The inferior survival result in SWOG 9712 compared to SWOG 9412 demonstrated that the addition of consolidation chemotherapy after CCRT led to increased toxicity without a survival benefit [13, 14]. Increased toxicities and uncertainty of a survival benefit of consolidation chemotherapy may result in the reluctance to prescribe and accept consolidation chemotherapy by oncologists and patients in our study.

With respect to the optimal chemotherapy regimen for ECOG 2 patients, the 3-year OS was much higher in the EP arm (37.5% vs. 7.5%) arm, though the OS did not reach the statistical difference. This might possibly due to the small sample size. The 3-year survival of ECOG 2 patients treated with EP regimen was comparable with good PS patients receiving CCRT reported in randomized clinical trials [15, 25]. In consistent with toxicity profile for our overall phase III trial population, more patients in the PC arm developed grade ≥ 3 radiation pneumonitis than those in EP arm (17.5% vs. 0%, *p* = 0.014). This was similar to the result of our previous phase II trial [26] and result of a meta-analysis of 836 patients reported by Palma et al. [27]. Treatment-related death were all due to grade 5 radiation pneumonitis in the PC arm. There was a trend that the incidence of grade 3 esophagitis was higher in the EP arm than in the PC arm (25.8% vs 10.0%, *p* = 0.078). The tolerability of concurrent chemoradiotherapy with EP was supported by the lower incidence of treatment related death and a higher percentage of patients in EP arm who completed concurrent chemotherapy as planned. With the development of immunotherapy, the NCCN guideline recommends durvalumab (category 1) as consolidation therapy for patients with stage III NSCLC who have not progressed after definitive concurrent chemoradiotherapy based on the PACIFIC trial. However, severe radiation pneumonitis from previous chemoradiotherapy was one of the contraindications of consolidation immunotherapy. As a result, the lower incidence of severe radiation pneumonitis in the EP arm may provide patients more chance to receive consolidation immunotherapy and thus contribute to prolonged survival.

The limitation of the study is that ECOG 2 subgroup analyses were not pre-planned in the phase III trial. The relatively small sample size of this subgroup may not be powered to make accurate inferences regarding the optimal chemotherapy regimen for the subsets. Moreover, except for ≥5% weight loss, the ECOG2 patients in our study had no other known poor prognostic factors listed above. Hence, these results should be interpreted with caution. Whether the results of the ECOG 2 subgroup analyses can be extrapolated to the real world ECOG2 population remains unclear.

## Conclusions

This prospective study demonstrates that ECOG 2 patients might benefit from CCRT with promising survival. Treatment discontinuation rate and toxicities were not significantly increased for ECOG 2 patients compared to those for ECOG 0–1 patients. For the ECOG 2 patients, the EP arm had similar survival compared to the PC arm. Compared with PC regimen, the EP regimen had a significantly lower incidence of grade ≥ 3 radiation pneumonitis and no fatal grade 5 radiation pneumonitis, thereby showing an acceptable safety profile in ECOG 2 patients. Prospective CCRT randomized study based on IMRT technique are warranted to validate our findings.

## Data Availability

The protocol and the datasets are available from the corresponding author on reasonable request.

## Abbreviations

ECOG: Eastern Cooperative Oncology Group
NSCLC: Non-small-cell lung cancer
LA-NSCLC: locally advanced non-small-cell lung cancer
CCRT: Concurrent chemoradiotherapy
3D-CRT: three-dimensional conformal radiation therapy
IMRT: Intensity-modulated radiation therapy
EP: Etoposide/cisplatin
PC: Carboplatin/paclitaxel
OS: Overall survival
FEV1: Forced expiratory volume in 1 s
RECIST: Response Evaluation Criteria in Solid Tumors
CTCAE: Common Toxicity Criteria for Adverse Events
PFS: Progression free survival
CSS: cancer specific survival
HR: Hazard ratios
CI: Confidence interval
SCC: Squamous cell carcinoma
GTV: Gross tumor volume
CR: Complete response
PR: Partial response
SD: Stable disease
SWOG: Southwest Oncology Group

## References

[1] Molina JR, Yang P, Cassivi SD, Schild SE, Adjei AA (2008). Non-small cell lung cancer: epidemiology, risk factors, treatment, and survivorship. Mayo Clin Proc.

[2] Yang P, Allen MS, Aubry MC, Wampfler JA, Marks RS, Edell ES, Thibodeau S, Adjei AA, Jett J, Deschamps C (2005). Clinical features of 5,628 primary lung cancer patients: experience at Mayo Clinic from 1997 to 2003. Chest.

[3] Chansky K, Sculier JP, Crowley JJ, Giroux D, Van Meerbeeck J, Goldstraw P (2009). The International Association for the Study of Lung Cancer staging project: prognostic factors and pathologic TNM stage in surgically managed non-small cell lung cancer. J Thoracic Oncology.

[4] Verger E, Salamero M, Conill C (1992). Can Karnofsky performance status be transformed to the Eastern Cooperative Oncology Group scoring scale and vice versa?. Eur J Cancer.

[5] Auperin A, Le Pechoux C, Rolland E, Curran WJ, Furuse K, Fournel P, Belderbos J, Clamon G, Ulutin HC, Paulus R (2010). Meta-analysis of concomitant versus sequential radiochemotherapy in locally advanced non-small-cell lung cancer. J Clin Oncol.

[6] Lilenbaum RC, Cashy J, Hensing TA, Young S, Cella D (2008). Prevalence of poor performance status in lung cancer patients: implications for research. J Thoracic Oncol.

[7] Ahn JS, Ahn YC, Kim JH, Lee CG, Cho EK, Lee KC, Chen M, Kim DW, Kim HK, Min YJ (2015). Multinational randomized phase III trial with or without consolidation chemotherapy using Docetaxel and Cisplatin after concurrent Chemoradiation in inoperable stage III non-small-cell lung Cancer: KCSG-LU05-04. J Clin Oncol.

[8] Senan S, Brade A, Wang LH, Vansteenkiste J, Dakhil S, Biesma B, Martinez Aguillo M, Aerts J, Govindan R, Rubio-Viqueira B (2016). PROCLAIM: randomized phase III trial of Pemetrexed-Cisplatin or Etoposide-Cisplatin plus thoracic radiation therapy followed by consolidation chemotherapy in locally advanced nonsquamous non-small-cell lung Cancer. J Clin Oncol.

[9] Bradley JD, Paulus R, Komaki R, Masters G, Blumenschein G, Schild S, Bogart J, Hu C, Forster K, Magliocco A (2015). Standard-dose versus high-dose conformal radiotherapy with concurrent and consolidation carboplatin plus paclitaxel with or without cetuximab for patients with stage IIIA or IIIB non-small-cell lung cancer (RTOG 0617): a randomised, two-by-two factorial phase 3 study. Lancet Oncol.

[10] Jegadeesh N, Liu Y, Gillespie T, Fernandez F, Ramalingam S, Mikell J, Lipscomb J, Curran WJ, Higgins KA (2016). Evaluating intensity-modulated radiation therapy in locally advanced non-small-cell lung Cancer: results from the National Cancer Data Base. Clin Lung Cancer.

[11] Liang J, Bi N, Wu S, Chen M, Lv C, Zhao L, Shi A, Jiang W, Xu Y, Zhou Z (2017). Etoposide and cisplatin versus paclitaxel and carboplatin with concurrent thoracic radiotherapy in unresectable stage III non-small cell lung cancer: a multicenter randomized phase III trial. Ann Oncol.

[12] Geskus RB (2011). Cause-specific cumulative incidence estimation and the fine and gray model under both left truncation and right censoring. Biometrics.

[13] Davies AM, Chansky K, Lau DH, Leigh BR, Gaspar LE, Weiss GR, Wozniak AJ, Crowley JJ, Gandara DR (2006). Phase II study of consolidation paclitaxel after concurrent chemoradiation in poor-risk stage III non-small-cell lung cancer: SWOG S9712. J Clin Oncol.

[14] Lau DH, Crowley JJ, Gandara DR, Hazuka MB, Albain KS, Leigh B, Fletcher WS, Lanier KS, Keiser WL, Livingston RB (1998). Southwest oncology group phase II trial of concurrent carboplatin, etoposide, and radiation for poor-risk stage III non-small-cell lung cancer. J Clin Oncol.

[15] Curran WJ, Paulus R, Langer CJ, Komaki R, Lee JS, Hauser S, Movsas B, Wasserman T, Rosenthal SA, Gore E (2011). Sequential vs. concurrent chemoradiation for stage III non-small cell lung cancer: randomized phase III trial RTOG 9410. J Natl Cancer Inst.

[16] Furuse K, Fukuoka M, Kawahara M, Nishikawa H, Takada Y, Kudoh S, Katagami N, Ariyoshi Y (1999). Phase III study of concurrent versus sequential thoracic radiotherapy in combination with mitomycin, vindesine, and cisplatin in unresectable stage III non-small-cell lung cancer. J Clin Oncol.

[17] Nawrocki S, Krzakowski M, Wasilewska-Tesluk E, Kowalski D, Rucinska M, Dziadziuszko R, Sowa A (2010). Concurrent chemotherapy and short course radiotherapy in patients with stage IIIA to IIIB non-small cell lung cancer not eligible for radical treatment: results of a randomized phase II study. J Thoracic Oncol.

[18] Strom HH, Bremnes RM, Sundstrom SH, Helbekkmo N, Flotten O, Aasebo U (2013). Concurrent palliative chemoradiation leads to survival and quality of life benefits in poor prognosis stage III non-small-cell lung cancer: a randomised trial by the Norwegian lung Cancer study group. Br J Cancer.

[19] Bi N, Wang L (2015). Superiority of concomitant chemoradiation over sequential chemoradiation in inoperable, locally advanced non-small cell lung cancer: challenges in the selection of appropriate chemotherapy. Semin Radiat Oncol.

[20] Semrau S, Bier A, Thierbach U, Virchow C, Ketterer P, Klautke G, Fietkau R (2007). 6-year experience of concurrent radiochemotherapy with vinorelbine plus a platinum compound in multimorbid or aged patients with inoperable non-small cell lung cancer. Strahlentherapie und Onkologie : Organ der Deutschen Rontgengesellschaft [et al].

[21] Steel GG, Hill RP, Peckham MJ (1978). Combined radiotherapy--chemotherapy of Lewis lung carcinoma. Int J Radiat Oncol Biol Phys.

[22] Harris JP, Murphy JD, Hanlon AL, Le QT, Loo BW, Diehn M (2014). A population-based comparative effectiveness study of radiation therapy techniques in stage III non-small cell lung cancer. Int J Radiat Oncol Biol Phys.

[23] Wang J, Zhou Z, Liang J, Feng Q, Xiao Z, Hui Z, Wang X, Lv J, Chen D, Zhang H (2016). Intensity-modulated radiation therapy may improve local-regional tumor control for locally advanced non-small cell lung Cancer compared with three-dimensional conformal radiation therapy. Oncologist.

[24] Chun SG, Hu C, Choy H, Komaki RU, Timmerman RD, Schild SE, Bogart JA, Dobelbower MC, Bosch W, Galvin JM (2017). Impact of intensity-modulated radiation therapy technique for locally advanced non-small-cell lung Cancer: a secondary analysis of the NRG oncology RTOG 0617 randomized clinical trial. J Clin Oncol.

[25] Hanna N, Neubauer M, Yiannoutsos C, McGarry R, Arseneau J, Ansari R, Reynolds C, Govindan R, Melnyk A, Fisher W (2008). Phase III study of cisplatin, etoposide, and concurrent chest radiation with or without consolidation docetaxel in patients with inoperable stage III non-small-cell lung cancer: the Hoosier oncology group and U.S. oncology. J Clin Oncol.

[26] Wang L, Wu S, Ou G, Bi N, Li W, Ren H, Cao J, Liang J, Li J, Zhou Z (2012). Randomized phase II study of concurrent cisplatin/etoposide or paclitaxel/carboplatin and thoracic radiotherapy in patients with stage III non-small cell lung cancer. Lung Cancer (Amsterdam, Netherlands).

[27] Palma DA, Senan S, Tsujino K, Barriger RB, Rengan R, Moreno M, Bradley JD, Kim TH, Ramella S, Marks LB (2013). Predicting radiation pneumonitis after chemoradiation therapy for lung cancer: an international individual patient data meta-analysis. Int J Radiat Oncol Biol Phys.

